# Shifting tuberculosis dynamics in the EU/EEA: geographical and drug resistance trends among people of foreign origin, 2019 to 2023

**DOI:** 10.2807/1560-7917.ES.2025.30.11.2500173

**Published:** 2025-03-20

**Authors:** Anca Vasiliu, Veronica Cristea, Krista Stoycheva, Senia Rosales-Klintz, Christoph Lange, Dominik Zenner, Csaba Ködmön

**Affiliations:** 1 Division of Clinical Infectious Diseases, Research Center Borstel, Borstel, Germany; 2 German Center for Infection Research (DZIF), Partner Site Hamburg-Lübeck-Borstel-Riems, Germany; 3 Department of Pediatrics, Global TB Program, Baylor College of Medicine, Houston, United States; 4 European Centre for Disease Prevention and Control (ECDC), Stockholm, Sweden; 5 Respiratory Medicine and International Health, University of Lübeck, Lübeck, Germany; 6 Wolfson Institute of Population Health, Barts and the London School of Medicine and Dentistry, Queen Mary University of London, London, United Kingdom

**Keywords:** tuberculosis, drug resistance, migrants, refugees, asylum seekers

## Abstract

We observed shifting trends in tuberculosis (TB) epidemiology in the EU/EEA between 2019 and 2023. In 2023, TB notifications among people of foreign origin increased by 24.6% after decreasing between 2019 and 2020. The majority originated from African and East Mediterranean regions, with a 316.4% upsurge in TB among Ukrainians in 2022–23. Rifampicin-resistant/multidrug-resistant TB increased by ≥ 60% in 2019–23, mainly among Ukrainians. Rapid response to shifting epidemiological patterns is crucial for efficient TB prevention and control within the EU/EEA.

Approximately one third of all people with tuberculosis (TB) in the European Union and European Economic Area (EU/EEA) are born in or have citizenship granted by a country other than the EU/EEA country notifying TB surveillance data to the European Centre for Disease Prevention and Control (ECDC). Recognising that migration to and within the EU/EEA has increased since 2015 [1,2] and has been further influenced by the COVID-19 pandemic (from 2020) and Russian invasion of Ukraine (from 2022), we wanted to assess the impact of those events on TB epidemiology in the EU/EEA. Previous findings underscore that the number of TB notifications among Ukrainians in the EU/EEA quadrupled between 2019 and 2022 following the invasion of Ukraine [3]. Here, we analysed geographical origin and drug resistance patterns among people of foreign origin with TB in the EU/EEA from 2019 to 2023, highlighting those of Ukrainian origin. 

## Geographical patterns

We used aggregated surveillance data of all TB notifications among people of foreign origin derived from case-based data notified to the European Surveillance System (TESSy) between 2019 and 2023. Case-based data from 30 EU/EEA countries were extracted from TESSy on 1 October 2024 (Liechtenstein did not report in 2019 and Latvia only reported data in 2021–22) and aggregated by regions of origin, according to the World Health Organization (WHO) classification [4]. We also differentiated between EU/EEA, non-EU/EEA countries and Ukraine within the WHO European Region. The modified United Nations’ M49 geoscheme was used for the European geographical grouping of the reporting country i.e. Western, Northern, Southern and Eastern Europe, with Cyprus included in the South European group [5]. The Supplement provides a detailed list of countries in each region of origin and European geographical grouping. We conducted a targeted analysis of people holding Ukrainian citizenship or born in Ukraine.

**Figure 1:**
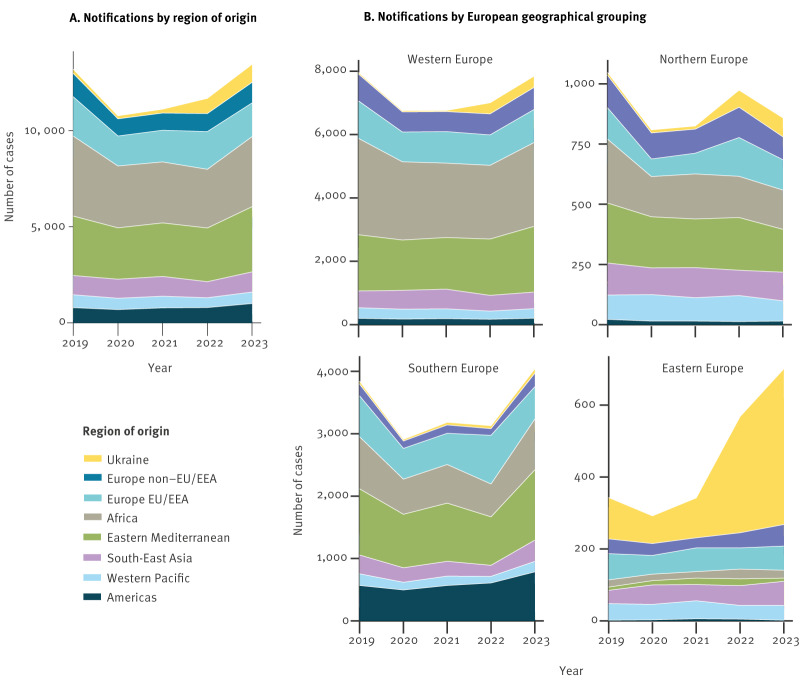
Number of tuberculosis notifications in people of foreign origin by (A) region of origin and by (B) European geographical grouping of reporting country, EU/EEA, 2019–2023 (n = 63,417)

The total number of TB notifications in people of foreign origin declined by 19% from 13,920 in 2019 to 11,271 in 2020, but increased by 24.6% to 14,049 notifications in 2023. The impact of the COVID-19 pandemic on travel restrictions, disruption in TB diagnostic and treatment services, and altered healthcare-seeking behaviour contributed to the reduction of notifications in 2020 [6,7]. By 2023, total numbers of TB notifications in people of foreign origin in the EU/EEA surpassed pre-pandemic levels (Figure 1A).

EU/EEA: European Union/European Economic Area.

Most people of foreign origin with TB were from outside the EU/EEA, predominantly from the African and Eastern Mediterranean regions (Figure 1A). The highest percent increase in TB notifications was observed in people of Ukrainian origin, with a substantial rise of 316.4% between 2019 and 2023. A smaller increase was observed among people from the Americas, with 27% rise in 2023 compared with 2019. Conversely, the number of people with TB from Western Pacific and non-EU European regions (except for Ukraine) decreased by 10.9% and 11.7%, respectively (Table).

**Table:** Number of tuberculosis notifications and yearly per cent change in people of foreign origin by region of origin and by European geographical grouping, EU/EEA, 2019–2023 (n = 63,417)

Grouping	2019	2020	% change 2020 vs 2019	2021	% change 2021 vs 2020	2022	% change 2022 vs 2021	2023	% change 2023 vs 2022	Overall % change 2023 vs 2019
European geographical grouping^a,b^										
Western Europe	7,968	6,763	−15.1	6,772	0.1	7,010	3.5	7,855	12.1	−1.4
Northern Europe	1,049	817	−22.1	832	1.8	987	18.6	865	−12.4	−17.5
Southern Europe	4,550	3,390	−25.5	3,679	8.5	3,976	8.1	4,620	16.2	1.5
Eastern Europe	353	300	−15.0	348	16.0	574	64.9	709	23.5	100.8
Total EU/EEA	13,920	11,270	−19.0	11,631	3.2	12,547	7.9	14,049	12.0	0.9
Region of origin overall										
Ukraine	226	161	−28.8	203	26.1	793	290.6	941	18.7	316.4
Europe EU/EEA	2,042	1,558	−23.7	1,647	5.7	1,960	19.0	1,755	−10.5	−14.1
Europe non-EU/EEA	1,210	893	−26.2	893	0.0	937	4.9	1,068	14.0	−11.7
Africa	4,158	3,216	−22.7	3,171	−1.4	3,051	−3.8	3,641	19.3	−12.4
Eastern Mediterranean	3,093	2,671	−13.6	2,784	4.2	2,793	0.3	3,388	21.3	9.5
South-East Asia	1,001	991	−1.0	1,028	3.7	834	−18.9	1,050	25.9	4.9
Western Pacific	668	588	−12.0	606	3.1	509	−16.0	595	16.9	−10.9
Americas	792	691	−12.8	780	12.9	797	2.2	1,006	26.2	27.0
In Western Europe^a^										
Ukraine	52	43	−17.3	36	−16.3	351	875	357	1.7	586.5
Europe non-EU/EEA	846	634	−25.1	631	−0.5	663	5.1	697	5.1	−17.6
Europe EU/EEA	1,183	940	−20.5	995	5.9	960	−3.5	1,043	8.6	−11.8
Africa	3,039	2,468	−18.8	2,347	−4.9	2,326	−0.9	2,643	13.6	−13.0
Eastern Mediterranean	1,774	1,589	−10.4	1,631	2.6	1,778	9.0	2,074	16.6	16.9
South-East Asia	530	594	12.1	621	4.5	497	−20	523	5.2	−1.3
Western Pacific	334	312	−6.6	309	−1.0	257	−16.8	302	17.5	−9.6
Americas	200	177	−11.5	191	7.9	171	−10.5	203	18.7	1.5
In Northern Europe^a^										
Ukraine	12	13	8.3	13	0.0	71	446.2	79	11.3	558.3
Europe non-EU/EEA	135	108	−20.0	100	−7.4	126	26.0	95	−24.6	−29.6
Europe EU/EEA	130	73	−43.8	86	17.8	161	87.2	126	−21.7	−3.1
Africa	265	167	−37.0	187	12.0	171	−8.6	163	−4.7	−38.5
Eastern Mediterranean	249	212	−14.9	202	−4.7	219	8.4	178	−18.7	−28.5
South-East Asia	133	111	−16.5	125	12.6	105	−16	119	13.3	−10.5
Western Pacific	101	110	8.9	97	−11.8	108	11.3	84	−22.2	−16.8
Americas	22	15	−31.8	15	0.0	13	−13.3	15	15.4	−31.8
In Southern Europe^a^										
Ukraine	47	28	−40.4	43	53.6	48	11.6	72	50.0	53.2
Europe non-EU/EEA	188	118	−37.2	134	13.6	106	−20.9	216	103.8	14.9
Europe EU/EEA	656	493	−24.8	500	1.4	780	56.0	519	−33.5	−20.9
Africa	834	563	−32.5	619	9.9	527	−14.9	813	54.3	−2.5
Eastern Mediterranean	1,061	858	−19.1	933	8.7	777	−16.7	1,127	45.0	6.2
South-East Asia	301	232	−22.9	237	2.2	177	−25.3	341	92.7	13.3
Western Pacific	187	123	−34.2	150	22.0	106	−29.3	168	58.5	−10.2
Americas	568	496	−12.7	568	14.5	608	7.0	786	29.3	38.4
In Eastern Europe^a^										
Ukraine	115	77	−33.0	111	44.2	323	191	433	34.1	276.5
Europe non-EU/EEA	41	33	−19.5	28	−15.2	42	50.0	60	42.9	46.3
Europe EU/EEA	73	52	−28.8	66	26.9	59	−10.6	67	13.6	−8.2
Africa	20	18	−10.0	18	0.0	27	50.0	22	−18.5	10.0
Eastern Mediterranean	9	12	33.3	18	50.0	19	5.6	9	−52.6	0.0
South-East Asia	37	54	45.9	45	−16.7	55	22.2	67	21.8	81.1
Western Pacific	46	43	−6.5	50	16.3	38	−24.0	41	7.9	−10.9
Americas	2	3	50.0	6	100	5	−16.7	2	−60.0	0.0

EU/EEA: European Union/European Economic Area.

^a^ Notifications are stratified by a European geographical grouping of reporting country. The Supplement provides a detailed list of countries in each region of origin and the European geographical grouping.

^b^ These data include TB notifications of ‘unknown origin’.

The analysis showed distinct patterns in the origin of foreign TB notifications depending on the European geographical grouping of reporting country (Figure 1B).

Western Europe experienced an overall rise in notifications from 2019 to 2023, with the largest absolute increase from Eastern Mediterranean (rising from 1,774 to 2,074). However, the highest percentage increase in this subregion was recorded among notifications from Ukraine (from 52 to 357; 586.5%). 

In contrast, notifications in people of foreign origin in Northern Europe decreased by 17.5% from 2019 to 2023. Africa and Eastern Mediterranean remained the leading regions of origin in terms of notifications, followed closely by non-EU/EEA European and South-East Asia regions. Tuberculosis notifications decreased among people of foreign origin in all regions compared with 2019, and numbers in the highest contributors (Eastern Mediterranean, African and non-EU/EEA European regions) have been decreasing in 2023 compared with 2022. TB among Ukrainian individuals increased from 12 in 2019 to 79 in 2023 (558.3%).

The number of TB notifications in people of foreign origin notified in Southern Europe fluctuated, with an increase primarily attributed to a surge in notifications from the Americas (38.4% rise since 2019) and the Eastern Mediterranean region. In 2023, the Eastern Mediterranean continued to account for most TB cases (n = 1,127), followed by Africa (n = 813). Notifications of TB among Ukrainian individuals increased by 53%, remaining low in absolute numbers (from 47 in 2019 to 72 in 2023).

In Eastern Europe, TB notifications in people of foreign origin increased since 2019, mainly driven by people of Ukrainian origin (from 115 in 2019 to 433 in 2023; 276.5%). TB notifications among people of foreign origin from other regions returned to pre-COVID-19 pandemic levels (Figure 1A).

## Drug resistance patterns

To analyse drug resistance patterns among TB cases in people of foreign origin, we used available data in TESSy for 30 countries on rifampicin-resistant (RR) and multidrug-resistant (MDR) TB, i.e. resistant to rifampicin and isoniazid. Additional national surveillance data regarding RR/MDR-TB were added to TESSy for France and Italy, as these countries shared data for pre-extensively drug-resistant (pre-XDR) and extensively drug-resistant (XDR) but had not updated TESSy for RR/MDR data. We used the term ‘drug-resistant’ for TB with resistance to any antibiotic used in TB treatment, as per WHO guidelines [8]. Our analysis focused on RR/MDR-TB, isoniazid mono-resistant TB, pre-XDR TB, i.e. RR/MDR-TB plus resistance to any fluoroquinolone, and XDR-TB, i.e. pre-XDR TB with additional resistance to either bedaquiline or linezolid [9].

During the study period, 67.9% (43,046/63,417) TB cases among people of foreign origin were laboratory-confirmed, i.e. positive identification of *Mycobacterium tuberculosis* complex confirmed by culture and/or presence of nucleic acid (range: 65.6% (9,134/13,920) in 2019; 71.3% (8,292/11,631) in 2021), while 64.9% (27,920/43,046) of laboratory-confirmed cases had a susceptibility test result available at least for rifampicin (range: 60.6% (5,022/8,292) in 2021; 68.0% (6,313/9,280) in 2023).

### Rifampicin-resistant/multidrug-resistant and isoniazid mono-resistant tuberculosis

We observed a slight increase in the proportion of RR/MDR-TB among all TB notifications in people of foreign origin in the EU/EEA from 2.2% (256/13,920) in 2019 to 3.7% (464/12,547) by 2022, followed by 3.5% (489/14,049) in 2023. This suggests that while RR/MDR-TB remains a concern, changes were modest.

**Figure 2:**
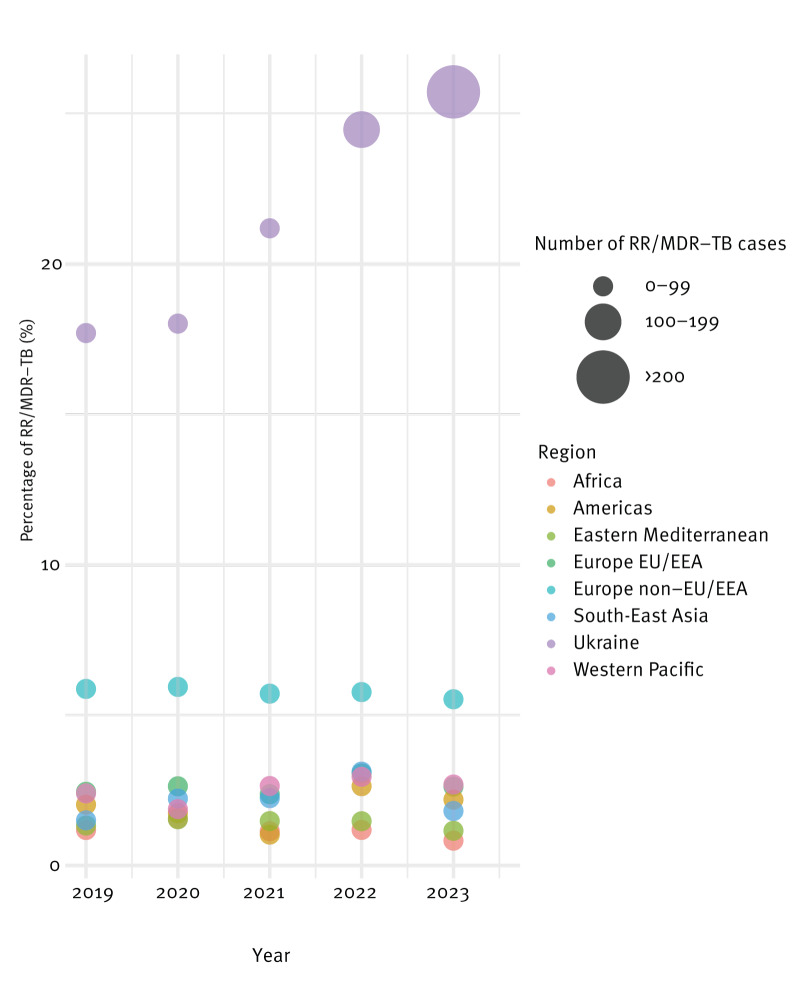
Rifampicin-resistant/multidrug-resistant tuberculosis among people of foreign origin per region of origin, EU/EEA, 2019–2023 (n = 1,804)

**Figure 3:**
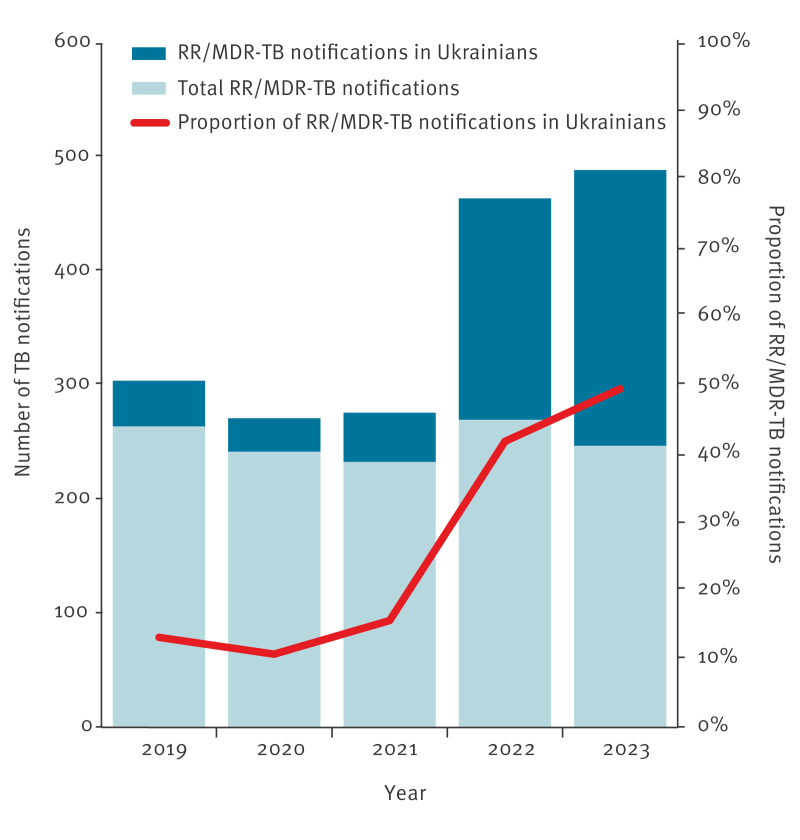
Rifampicin-resistant/multidrug-resistant tuberculosis notifications among people of foreign origin overall and with Ukrainian origin, EU/EEA, 2019–2023 (n = 1,804)

Analysing the data by region of origin demonstrated that RR/MDR-TB among people of Ukrainian origin increased from 17.7% (40/226) in 2019 to 25.7% (242/941) in 2023 (Figure 2). Contrarily, the proportion of RR/MDR-TB notifications in people originating from non-EU/EEA European countries except for Ukraine remained stable at around 6% during the study period. The other regions of origin showed rates between 1.5% and 2.4% (Figure 2). Notably, by 2023, TB notifications in people of Ukrainian origin represented 49.5% of all RR/MDR notifications among people of foreign origin (Figure 3).

EU/EEA: European Union/European Economic Area; MDR: multidrug-resistant; RR: rifampicin-resistant.

The y-axis represents the percentage of RR/MDR-TB notifications among all notifications in people of foreign origin diagnosed with TB. The size of the bubbles represents the number of RR/MDR-TB notifications.

EU/EEA: European Union/European Economic Area; RR/MDR: rifampicin-resistant/multidrug-resistant; TB: tuberculosis.

Dark blue bars: RR/MDR-TB notifications in people of Ukrainian origin (n = 548). Light blue bars: total RR/MDR-TB notifications among people of foreign origin overall (n = 1,256). Red line: proportion of RR/MDR-TB notifications in people of Ukrainian origin among overall RR/MDR-TB notifications of foreign origin (right side Y-axis).

The overall percentage of monoresistance to isoniazid in TB cases of foreign origin had a gradual upward trajectory, rising from 4.5% (623/13,920) in 2019 to 6.7% (840/12,547) in 2022, before stabilising in 2023 at 5.9% (830/14,049).

### Pre-extensively drug-resistant and extensively drug-resistant tuberculosis

The proportion of pre-XDR among TB notifications in people of foreign origin in 2019 was 0.3%, with 42 notified cases, doubling in 2022 and 2023 (0.6%), reaching 86 notified cases in 2023, 52 of which originated from Ukraine.

During the period 2019–23, 17 XDR-TB cases in people of foreign origin were reported, accounting for 0.03% of all TB notifications. With one case reported in 2019 and 2021 and no cases in 2020. Numbers doubled from 2022 (n = 5) to 2023 (n = 10) with Ukrainians accounting for a fourfold increase (n = 1 in 2022 vs n = 5 in 2023), reaching half of XDR-TB notifications in people of foreign origin in 2023.

## Discussion

This analysis highlights the rising trend in TB and RR/MDR-TB notifications among people of foreign origin in the EU/EEA, with Western Europe showing the highest numbers. This increase of TB and drug-resistant TB [1] is driven by the displacement of over 5 million people during the Russian war against Ukraine [10], which contributed to doubling the proportion of pre-XDR-TB cases, where 60% were in people of Ukrainian origin. Tuberculosis notifications in people from the African and Eastern Mediterranean regions show the highest absolute numbers in the EU/EEA, as previously highlighted [11]; however, RR/MDR-TB rates from these regions remain low (< 3%). Nevertheless, given the high costs of RR/MDR-TB regimens, issues related to procurement and funding of drug-resistant TB treatment in the EU/EEA should be addressed [12].

There are several limitations, including the fact that the definitions of pre-XDR-TB and XDR-TB have changed during the study period [9]. A sub-optimal drug-resistant testing coverage was observed during the study period alongside the incompleteness of resistance data reported by EU/EEA countries to TESSy [9]. We mitigated these limitations by using the latest definitions of pre-XDR and XDR [9] and by complementing the resistance information from the countries with missing data in TESSy to ensure data completion for this analysis. In addition, case notifications depend on testing capacity in EU/EEA, which is not fully available for all the drugs used to treat RR/MDR-TB [13]. Therefore, this analysis reflects the resistance proportions among the tested populations. Another data-related limitation is that some cases could be counted more than once, if the same case is reported from different EU/EEA countries. Other limitations are the lack of age and sex variables in aggregated format and the lack of denominator data for incidence calculation. While incidence rates provide crucial information for clinical risk assessment, this was not the objective of our current analysis.

## Conclusions

By 2023, TB notifications in people of foreign origin returned to pre-pandemic levels in the EU/EEA. Notifications among people of Ukrainian origin increased substantially during the study period, similar for drug-resistant TB where increases of ≥ 60% were observed. Rapid adaptation to shifting epidemiological patterns, increasing availability of drug susceptibility data, improved drug resistance surveillance, better follow-up of mobile populations and strengthening public health response is crucial to control TB spread within the EU/EEA. 

## Supplementary Data

44000 Supplement
