# Supplementary material for: Shifting tuberculosis dynamics in the EU/EEA: geographical and drug resistance trends among people of foreign origin, 2019 to 2023
Source: Euro Surveill. 2025 Mar 20;30(11):2500173. doi: 10.2807/1560-7917.ES.2025.30.11.2500173 (PMC11927073; doi:10.2807/1560-7917.ES.2025.30.11.2500173)
Supplement: Supplement [file 25-00173_VASILIU_Supplement.pdf]

Tuberculosis shifting dynamics in the EU/EEA: geographical and drug resistance trends among persons of foreign origin, 2019-2023

This supplementary material is hosted by Eurosurveillance as supporting information alongside the article "Tuberculosis shifting dynamics in the EU/EEA: geographical and drug resistance trends among persons of foreign origin, 2019-2023" on behalf of the authors who remain responsible for the accuracy and appropriateness of the content. The same standards for ethics, copyright, attributions and permissions as for the article apply. Eurosurveillance is not responsible for the maintenance of any links or email addresses provided therein.

Supplementary material

Countries/areas by WHO region

WHO African Region
Algeria, Angola, Benin, Botswana, Burkina Faso, Burundi, Cabo Verde, Cameroon, Central African Republic, Chad, Comoros, Congo, Côte d'Ivoire, Democratic Republic of the Congo, Equatorial Guinea, Eritrea, Eswatini, Ethiopia, Gabon, Gambia, Ghana, Guinea, Guinea-Bissau, Kenya, Lesotho, Liberia, Madagascar, Malawi, Mali, Mauritania, Mauritius, Mozambique, Namibia, Niger, Nigeria, Rwanda, Sao Tome and Principe, Senegal, Seychelles, Sierra Leone, South Africa, South Sudan, Togo, Uganda, United Republic of Tanzania, Zambia, Zimbabwe.

WHO Region of the Americas
Antigua and Barbuda, Argentina, Bahamas, Barbados, Belize, Bolivia (Plurinational State of), Brazil, Canada, Chile, Colombia, Costa Rica, Cuba, Dominica, Dominican Republic, Ecuador, El Salvador, Grenada, Guatemala, Guyana, Haiti, Honduras, Jamaica, Mexico, Nicaragua, Panama, Paraguay, Peru, Puerto Rico (*Associate WHO Member State), Saint Kitts and Nevis, Saint Lucia, Saint Vincent and the Grenadines, Suriname, Trinidad and Tobago, United States of America, Uruguay, Venezuela (Bolivarian Republic of).

WHO South-East Asia Region
Bangladesh, Bhutan, Democratic People's Republic of Korea, India, Indonesia, Maldives, Myanmar, Nepal, Sri Lanka, Thailand, Timor-Leste.

WHO European Region
Albania, Andorra, Armenia, Austria, Azerbaijan, Belarus, Belgium, Bosnia and Herzegovina, Bulgaria, Croatia, Cyprus, Czechia, Denmark, Estonia, Finland, France, Georgia, Germany, Greece, Hungary, Iceland, Ireland, Israel, Italy, Kazakhstan, Kyrgyzstan, Latvia, Lithuania, Luxembourg, Malta, Monaco, Montenegro, Netherlands, North Macedonia, Norway, Poland, Portugal, Republic of Moldova, Romania, Russian

Federation, San Marino, Serbia, Slovakia, Slovenia, Spain, Sweden, Switzerland, Tajikistan, Türkiye, Turkmenistan, Ukraine, United Kingdom of Great Britain and Northern Ireland, Uzbekistan.

WHO Eastern Mediterranean Region
Afghanistan, Bahrain, Djibouti, Egypt, Iran (Islamic Republic of), Iraq, Jordan, Kuwait, Lebanon, Libya, Morocco, Oman, Pakistan, Qatar, Saudi Arabia, Somalia, Sudan, Syrian Arab Republic, Tunisia, United Arab Emirates, West Bank and Gaza Strip (*Non-Member area), Yemen.

WHO Western Pacific Region
Australia, Brunei Darussalam, Cambodia, China, Cook Islands, Fiji, Japan, Kiribati, Lao People's Democratic Republic, Malaysia, Marshall Islands, Micronesia (Federated States of), Mongolia, Nauru, New Zealand, Niue, Palau, Papua New Guinea, Philippines, Republic of Korea, Samoa, Singapore, Solomon Islands, Tokelau (*Associate WHO Member State), Tonga, Tuvalu, Vanuatu, Viet Nam.

United Nations geoscheme for Europe* used to group EU/EEA countries in geographical groups:

Western Europe
Austria, Belgium, France, Germany, Liechtenstein, Luxembourg, Netherlands (Kingdom of the), Switzerland.

Northern Europe
Denmark, Estonia, Finland, Iceland, Ireland, Latvia, Lithuania, Norway, Sweden.

Southern Europe
Albania, Andorra, Bosnia and Herzegovina, Cyprus, Croatia, Greece, Italy, Malta, Montenegro, North Macedonia, Portugal, Serbia, Slovenia, Spain.

Eastern Europe
Bulgaria, Czechia, Hungary, Poland, Romania, Slovakia.

*San Marino and Monaco have been excluded from the geoscheme as they are not part of the EU/EEA
